# Substance Use and Mental Health in Emerging Adult University Students Before, During, and After the COVID-19 Pandemic in Mexico: A Comparative Study

**DOI:** 10.3390/diseases12120303

**Published:** 2024-11-27

**Authors:** Gustavo A. Hernandez-Fuentes, Jessica C. Romero-Michel, Veronica M. Guzmán-Sandoval, Janet Diaz-Martinez, Osiris G. Delgado-Enciso, Ruth R. Garcia-Perez, Monserrat Godínez-Medina, Vicente Zamora-Barajas, Angel G. Hilerio-Lopez, Gabriel Ceja-Espiritu, Mario Del Toro-Equihua, Margarita L. Martinez-Fierro, Idalia Garza-Veloz, Iram P. Rodriguez-Sanchez, Carmen A. Sanchez-Ramirez, Mario Ramirez-Flores, Ivan Delgado-Enciso

**Affiliations:** 1 Department of Molecular Medicine, School of Medicine, University of Colima, Colima 28040, Mexico; gahfuentes@gmail.com (G.A.H.-F.); 1933osiris@gmail.com (O.G.D.-E.); ruthrubi_garcia@ucol.mx (R.R.G.-P.); mgodinez4@ucol.mx (M.G.-M.); vzamora4@ucol.mx (V.Z.-B.); gcejae11@ucol.mx (G.C.-E.); mequihua@ucol.mx (M.D.T.-E.); carmen_sanchez@ucol.mx (C.A.S.-R.); mario_ramirez@ucol.mx (M.R.-F.); 2 Faculty of Law, University of Colima, Colima 28040, Mexico; jessica_romero@ucol.mx; 3 Faculty of Psychology, University of Colima, Colima 28040, Mexico; gus_vero@ucol.mx; 4 Research Center in a Minority Institution, Florida International University (FIU-RCMI), Miami, FL 33199, USA; jdimarti@fiu.edu; 5 Faculty of Nursing, University of Colima, Colima 28040, Mexico; ahilerito@ucol.mx; 6 Molecular Medicine Laboratory, Unidad Académica de Medicina Humana y Ciencias de la Salud, Universidad Autónoma de Zacatecas, Zacatecas 98160, Mexico; margaritamf@uaz.edu.mx (M.L.M.-F.); idaliagv@uaz.edu.mx (I.G.-V.); 7 Molecular and Structural Physiology Laboratory, School of Biological Sciences, Universidad Autónoma de Nuevo León, San Nicolás de los Garza 66455, Mexico; iramrodriguez@gmail.com; 8 State Cancerology Institute of Colima, Health Services of the Mexican Social Security Institute for Welfare (IMSS-BIENESTAR), Colima 28085, Mexico; 9 Robert Stempel College of Public Health and Social Work, Florida International University, Miami, FL 33199, USA

**Keywords:** COVID-19, depression, anxiety, university students, substance use, tobacco and alcohol consumption

## Abstract

Background: The COVID-19 pandemic significantly impacted mental health and substance use patterns, particularly among young adults. Objective: This study aimed to assess changes in anxiety, depression, self-esteem, and substance use among university students in Mexico before, during, and after the pandemic. Methods: Using a repeated cross-sectional design, this study was conducted with university students in Mexico across three periods: pre-pandemic (2017 and 2019); during the pandemic (2021); and post-pandemic (2023). A total of 2167 students were interviewed during one of the three periods. Standardized scales measured anxiety, depression, self-esteem, Erotic Response and Sexual Orientation Scale (EROS), and substance use. Results: showed a marked and significant increase in the proportion of students with anxiety (40.0%, 71.7%, and 79.6%) and depression (14.4%, 61.9%, and 62.6%) during the pre-pandemic, pandemic, and post-pandemic periods, respectively. Self-esteem significantly decreased during and after the pandemic, compared to pre-pandemic, particularly among females. The proportion of students categorized as moderate/high-risk for their substance use changed over time, showing a reduction in alcohol use (from 29.9% to 20.2%) and tobacco use (from 26.0% to 18.2%) but an increase in sedative use (from 7.1% to 11.7%), before vs after the pandemic, respectively. Multivariate analysis revealed that anxiety, low self-esteem, and increased sedative use were consistently linked to a heightened risk of depression during and after the pandemic. Notably, anxiety and depression levels remained in a proportion significantly elevated even in the post-pandemic period. Conclusions: These findings underscore the enduring impact of the COVID-19 pandemic on the mental health of university students, highlighting the urgent need for targeted interventions, early detection strategies, and customized educational programs to effectively support students’ mental well-being in the ongoing post-pandemic era.

## 1. Introduction

The COVID-19 pandemic has posed an unprecedented challenge to global health, profoundly impacting various aspects of daily life, including mental health. This impact has been particularly significant among emerging adult university students, a group already showing vulnerability before the pandemic. Numerous studies have documented a notable increase in levels of anxiety, depression, and other mental disorders during this period. For example, research conducted in Mexico during the lockdown revealed a 30% increase in depressive symptoms among university students compared to pre-pandemic data [1,2]. These findings align with international studies, suggesting a widespread deterioration in young people’s mental health related to social isolation, economic uncertainty, and educational disruptions, among other unknown factors [3,4].

Before the pandemic, the prevalence of anxiety and depression disorders among emerging adults in Mexico was already concerning, with rates ranging from 19% to 31% for anxiety and 12% to 25% for depression [5,6]. The pandemic not only exacerbated these problems but also highlighted the unpreparedness of mental health systems to meet the growing demand for psychological support. Additionally, access to mental health services was affected, leading many young people to resort to self-medication and substance use to cope with stress [7,8].

However, most studies have focused on the pandemic’s impact during the lockdown period without adequately exploring how these mental health issues evolved before and after the pandemic. Some longitudinal studies made during the pandemic time showed that anxiety and depression levels decreased over time [9]. It is also crucial to consider social differences as a significant factor in the experience of the pandemic and its aftermath. Socioeconomic inequalities have played an important role in increasing vulnerability to mental health issues. For instance, young people from disadvantaged socioeconomic backgrounds reported higher levels of stress, anxiety, and depression during the pandemic [10]. Similar patterns of inequality and mental health have been observed in other countries that have conducted comparable research, such as Brazil, Spain, and the United States of America [5,9,11,12].

Furthermore, substance use disorders have been a significant problem affecting health, including mental health, safety, and overall well-being. Stigma and discrimination reduce the likelihood of people at risk due to substance use receiving the necessary support. Fewer than 20% of people with these disorders receive treatment, and the availability of treatment is highly inconsistent. Although nearly half of amphetamine-type stimulant users are female, only 27% of those needing treatment are in treatment programs [13,14]. This study will also explore how substance use has changed in this population before, during, and after the pandemic.

Regarding substance use, previous studies have shown an increase in alcohol and tranquilizer consumption among young people during the pandemic, while tobacco and other drug use varied depending on social context and restrictions implemented [15,16,17,18]. However, there is a lack of comparative studies analyzing these changes over time, especially in Latin American countries like Mexico, where cultural and socioeconomic differences may influence how young people cope with crises.

Regarding the instruments employed in previous research, a variety of instruments such as the Beck Depression Inventory (BDI) [19], Generalized Anxiety Disorder 7 (GAD-7) [20], and the Rosenberg Self-Esteem Scale (RSES) [21] have been commonly used to assess mental health indicators among young adults. Additionally, surveys on substance use have utilized standardized questionnaires like the Alcohol Use Disorders Identification Test (AUDIT) [22] and the Drug Use Screening Inventory (DUSI) [23]. Although the use of these instruments is subjective and does not indicate a diagnostic value per se, they are valid tools for obtaining paradigms and trends in the population, especially when comparison values are available [24], as in this study before, during, and after the pandemic.

The aim of this study is to analyze trends in levels of anxiety, depression, self-esteem, and substance use among emerging adult university students in Mexico before, during, and after the COVID-19 pandemic. This analysis seeks to provide insights that could inform the development of educational, health, and psychological care strategies aimed at establishing new approaches to support this population.

## 2. Materials and Methods

### 2.1. Study Design and Subjects

For this study, a repeated cross-sectional design (RCS) was chosen to effectively capture mental health and addiction variables in university student groups across three different time periods (before, during, and after the COVID-19 pandemic restrictions) [25]. An RCS analyzes a collection of individual-level data repeated at regular intervals, where the same individuals are not necessarily included in each analyzed period. This design was selected because it is particularly useful when there is a dynamic component in this study, allowing for the investigation of relationships that change over time [26]. The time elapsed between the pre- and post-pandemic restriction periods was four years or more, during which students could have graduated from the university, making this a dynamic component that influenced the choice of an RCS instead of a cohort design. Additionally, the learning environments for students (in-person or online) varied dramatically during the pandemic due to educational policy changes [27,28]. These factors necessitated a flexible design capable of providing insights into student experiences under rapidly changing conditions. This study was reported in accordance with the STROBE guidelines for the presentation of observational studies [29]. This study included emerging adult students from the University of Colima, located in a city of approximately 400,000 inhabitants in western Mexico. Participants were enrolled in various undergraduate programs, including medicine, nursing, nutrition, law, mechatronics, architecture, and business administration. This study was approved by the Research Ethics Committee of the State Cancer Institute and university authorities (approval number CEICANCL230317-ASEXCCC-06, 2 March 2017). Participants were required to give informed consent, specifying the voluntary nature of the survey, with confidentiality guaranteed, as no identifying data were collected. Information was gathered from three time periods: before the pandemic (October–December 2017 and October–December 2019); during pandemic restrictions with distance education and online classes (October–December 2021); and one year after the resumption of unrestricted in-person educational activities (November–December 2023). This last period was considered “after the pandemic”, and although technically the pandemic is still in effect, the emergency, isolation, and restrictions had already ended. Inclusion criteria included university students enrolled in in-person educational programs, aged 17 to 25 years, who voluntarily agreed to participate in this survey. Students with a prior diagnosis of psychiatric illness were excluded. Additionally, data were screened for missing or anomalous entries. Participants with incomplete responses or inconsistent data patterns were excluded from this analysis. For those with partially missing data, the method of multiple imputation was used, ensuring that the sample size remained consistent while accounting for potential biases introduced by nonresponse [30]. Any records with more than 50% missing data were excluded entirely from this analysis. A non-probabilistic sampling method was used, and students were invited to participate. This survey was conducted in person during school hours in 2017 and 2019, while it was conducted online in 2021 and 2023.

### 2.2. Procedure

Anxiety and depression levels were assessed using the Spanish version of the Hospital Anxiety and Depression Scale (HADS), which consists of 11 items and two subscales: the depression scale with six items and the anxiety scale with five items [31]. Each item has four possible responses, scored from 0 to 3, for a total of 0 to 21 for depressive symptoms and 0 to 21 for anxiety. Clinical cut-off points were established at ≥8 for anxiety and ≥7 for depression, as previously reported [31,32]. The presence of anxiety or depression was categorized as an independent variable, and it was the measure by which the students were divided into cases and controls. Differences were evaluated between the pre-pandemic period (years 2017 and 2019), the period during the pandemic with mobility restrictions and online classes (year 2021), and the period without mobility restrictions after a year of “normality” in educational processes.

General variables such as age, gender, a program of study, semester, weight, and height (for calculating body mass index) were collected. Additionally, students were asked if they had engaged in vaginal or anal sexual intercourse (defined as penetration of the penis into the vagina or anus), the age at which this occurred, and the number of sexual partners they had to date. This information was collected because sexual activity has been shown to be associated with mental health outcomes such as anxiety and depression in emerging adults, a key developmental phase marked by relationship-building and identity formation [33,34,35]. The Erotic Response and Sexual Orientation Scale (EROS) was used to assess erotophilia (positive attitudes toward sexual stimuli), as these factors could influence emotional health. Prior studies suggest that sexual attitudes may affect anxiety and depression levels, particularly in young adults [36]. An adapted Spanish version of the Erotic Response and Sexual Orientation Scale (EROS) was also used [37,38]. The total possible punctuation was 0 (maximal erotophobia) to 120 (maximal erotophilia). A score of 60 or more was considered positive (erotophilia).

The socioeconomic level was categorized based on the 2022 guidelines of the Mexican Association of Market Intelligence and Opinion Agencies (AMAI) [39,40]. Households were classified into three groups: A/B (high); C (middle level); and D/E (vulnerable middle-class/poor) [39,41,42]. Automobile ownership was also recorded as a proxy for socioeconomic status (SES), an important determinant of mental health [43,44]. In Mexico, car ownership is often indicative of material wealth and access to resources [45]. Additionally, previous studies have demonstrated that car ownership correlated with increased sexual activity among young adults, possibly due to the privacy and mobility it offers [45]. By including car ownership, we aim to assess both its role as an SES indicator and its association with sexual behavior, which may influence mental health [43,44]. The self-esteem of emerging adults was assessed using the Rosenberg Scale, which has been adapted and validated in Spanish [46,47,48,49]. The Rosenberg Scale has a score range from 10 to 40, with low self-esteem classified as a score equal to or lower than 29 [45,50]. 

Additionally, the Alcohol, Smoking, and Substance Involvement Screening Test (ASSIST), developed by the World Health Organization (WHO), was administered and adapted for Spanish-speaking populations [51,52]. Patients were identified as being at moderate/high risk (moderate risk of health and other problems due to current substance use patterns; high risk are those subjects with a high probability of experiencing serious health, social, financial, legal, or relationship problems because of current use patterns and are likely to be dependent) [53,54].

In the surveys conducted in 2021 and 2023, participants were asked if they had contracted COVID-19, if any family members living in their household had died from the disease, and the COVID-19-Related Worry Scale was used as a proxy for perceived stress related to the pandemic. This scale assesses COVID-19-related worry with a 1–10 numerical rating scale, determined by the response to the following question: “On a scale of one to ten, how worried are you about the COVID-19 pandemic? One being not worried at all, and ten being extremely worried” [55].

### 2.3. Sample Size

A previous study conducted among students in Finland reported that anxiety in some subgroups of young people increased from 19.5% (before the pandemic) to 29.8% during the pandemic [56]. Based on these data, a sample size of 274 subjects before and during the pandemic was calculated to detect differences, with a power of 80% (α = 0.05).

### 2.4. Statistical Analysis

Data are presented as percentages or means and their standard deviations. The normality of the data was assessed using Kolmogorov–Smirnov tests. To compare three groups (intergroup analysis), ANOVA or Fisher’s exact tests were used for quantitative or qualitative data, respectively. Comparisons between the two groups used the independent Student’s *t*-test or Fisher’s exact test. To determine the association between the presence of depression or anxiety and various risk factors, multivariate binary logistic regression analysis was performed to obtain adjusted odds ratios (AdOR) with their 95% confidence intervals (CI) and *p*-values. Variables for the most parsimonious multivariate model were selected using the backward stepwise selection method. The probability used for stepwise regression was set at 0.15 for variable entry and 0.25 for elimination. Data were analyzed using SPSS Statistics version 20 software (IBM Corp., Armonk, NY, USA) [57]. Sample size calculations were performed using the online software ClinCalc version 1 (https://clincalc.com/stats/Power.aspx; accessed on 10 May 2024). A *p* < 0.05 was considered statistically significant [58].

## 3. Results

A total of 1179 students were included and analyzed before the pandemic, 779 during, and 209 after (see Table 1). Across all three periods, 148 students either refused to participate in this study or did not meet the inclusion criteria. Notably, the average age was 19.7 ± 1.4 years, with 65.1% of the participants being female (see Table 1).

**Figure 1:**
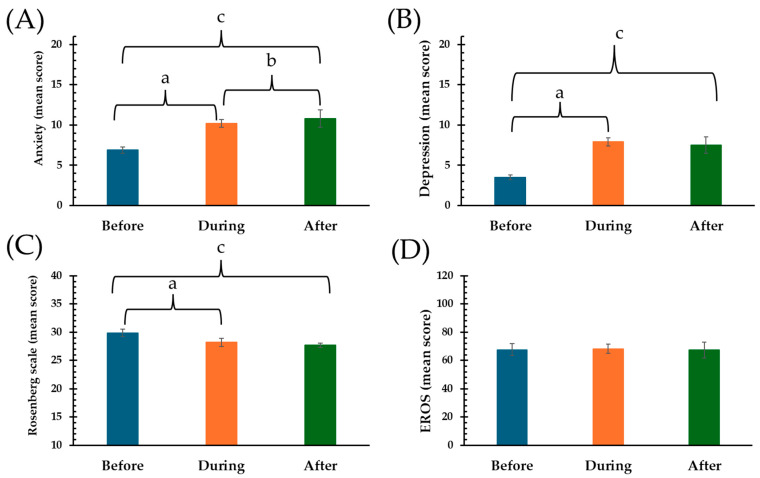
Mean scores of anxiety, depression, Rosenberg Self-Esteem Scale, and EROS before, during, and after the COVID-19 pandemic presented as mean and 95% confidence intervals (CI). (**A**) Hospital Anxiety. (**B**) Depression Scale (HADS) has a score range of 0 to 21 for depressive symptoms and 0 to 21 for anxiety, with clinical cut-off points of ≥8 for anxiety and ≥7 for depression. (**C**) The Rosenberg Self-Esteem Scale scores range from 10 to 40, with low self-esteem classified as a score of 29 or lower. (**D**) EROS (Erotic Response and Sexual Orientation Scale) indicates a score of 60 or higher for erotophilia. Statistical differences were determined at *p* > 0.05 as follows: ^a^ Before vs. During; ^b^ During vs. After; and ^c^ Before vs. After.

The average levels of anxiety and depression (HADS score) were 6.9 and 3.5, respectively, before the pandemic. There was a noticeable increase in anxiety (10.2) and depression (7.9) scores during the pandemic, which remained elevated after the pandemic (10.8 for anxiety and 7.5 for depression) (see Figure 1 and Table 2). Post-pandemic depression levels showed a similarity to those during the pandemic (*p* = 0.273), whereas anxiety levels exhibited an increase after the pandemic (*p* = 0.043). The self-esteem scores were lower during and after the pandemic compared to the pre-pandemic period (*p* < 0.001 for all comparisons). The Erotic Response and Sexual Orientation Scale scores showed no significant differences between periods. These scores were used to dichotomize subjects to identify those with anxiety, depression, low self-esteem, and erotophilia (see Table 3). The proportion of young adults with anxiety increased from 40.0% before the pandemic to 71.7% during the pandemic and further to 79.6% after the pandemic. Depression increased from 14.4% before the pandemic to 61.9% during the pandemic and remained high after the pandemic (62.6%). Low self-esteem was present in 48.5% of students before the pandemic, increasing to 57.9% during the pandemic and 66.5% after the pandemic. Erotophilia was present in 65% of the young adults and did not change across the periods analyzed (*p* = 0.760, intergroup Fisher’s exact test) (see Table 3).

Users with a low risk of substance-related problems scored three or less (or ten or less for alcohol). While they may occasionally use substances, their current consumption habits indicate a low likelihood of developing future issues. In contrast, those with moderate risk scoring between 4 and 26 (or 11 to 26 for alcohol) might already experience some challenges and are at a moderate risk of facing health and other related problems. Continuing to consume substances at this level suggests a growing likelihood of future health complications, including dependence. This risk is even greater for users with a history of substance abuse or dependence. A high-risk category is assigned to individuals scoring 27 or higher for any substance, indicating a strong possibility of dependence and likely existing problems related to health, social interactions, economics, legal matters, or personal relationships due to substance use. Furthermore, those who have injected drugs more than four times per month in the last three months are also considered at high risk. Additionally, Table 3 shows the proportion of young adults with moderate/high risk for various addictions (according to the ASSIST) [52]. Alcohol was the most consumed substance (moderate/high risk), with a prevalence of 29.9% before the pandemic, significantly decreasing to 26.2% and 20.2% during and after the pandemic, respectively (*p* < 0.05 for both comparisons). Moderate/high risk by tobacco was 26.0%, decreasing to 19.6% and 18.2% during and after the pandemic, respectively (*p* < 0.05 for both comparisons). Conversely, the use of sedatives or sleeping pills increased after the pandemic compared to the period before the pandemic (*p* = 0.034) (7.1%, 8.1%, and 11.7% before, during, and after the pandemic). The risk of consuming other substances did not show significant changes between periods.

In addition to the previously mentioned findings, Table 3 allows for an analysis of how the pandemic differentially impacted young people based on the nature of their addictions and behaviors. It is noteworthy that, despite the overall decrease in moderate/high risk for alcohol and tobacco consumption, not all addictions followed this trend. The increase in the use of tranquilizers or sleeping pills is associated with changes in coping mechanisms for stress and anxiety, which may relate to emotional difficulties experienced during periods of confinement and uncertainty.

Another important observation is that while some patterns of consumption of some substances decreased during the pandemic, the risk of consuming illicit substances such as marijuana, cocaine, amphetamines, and hallucinogens remained relatively stable across all periods. There appears to be a relationship between the reduction in consumption of accessible substances like alcohol and tobacco among young people and the observed stability in behaviors among those at risk for illicit drug use, suggesting that underlying factors remained consistent throughout the pandemic.

Table 3 highlights a potential gap in access to or willingness to seek support or treatment for addictions, particularly in the context of the pandemic, where mental health and addiction services may have been limited. The stability or increase in certain addictions may be associated with an unmet need for psychological and social support for young people during and after the pandemic.

The socioeconomic level did not significantly modify the proportion of students experiencing anxiety before or during/after the pandemic (see Table 4). However, during/after the pandemic, depression was less prevalent among students from high socioeconomic backgrounds (53.4%) compared to those from middle (67.6%, *p* < 0.001) and low (64.2%, *p* < 0.001) socioeconomic backgrounds.

Car ownership reduced the proportion of students with anxiety and depression both before and during/after the pandemic (see Table 4), with the greatest impact observed on the reduction in depression during/after the pandemic. In this period, 65.1% of students without a car experienced depression, compared to 45.8% of students with a car (*p* < 0.001). Factors such as socioeconomic level could modify access to services and resources that influenced mental health, which became particularly relevant during/after the pandemic. For instance, prior to the pandemic, car ownership was higher among students from high socioeconomic backgrounds (41.9%) compared to those from middle (22.3%, *p* = 0.008) or low (18.4%, *p* = 0.009) backgrounds. During/after the pandemic, car ownership was also greater among students from high socioeconomic backgrounds (25.1%) compared to those from middle (10.2%, *p* < 0.001) or low (9.2%, *p* < 0.001) backgrounds.

In Table 5, it is possible to observe how sex differences manifest in various psychological and behavioral aspects during different phases of the pandemic. Table 5 shows that females experienced a more significant increase in anxiety and depression compared to males, especially during and after the pandemic. Together, these tables illustrate how the pandemic differently impacted mental health and addiction risks across the sexes. While overall addiction risks may have declined, the sharp increase in mental health issues among females suggests that the stress and challenges of the pandemic may have been disproportionately felt by this group. This underscores the importance of considering sex-specific approaches in both mental health interventions and addiction prevention strategies to address the unique needs of each group during and after such crises.

**Figure 2:**
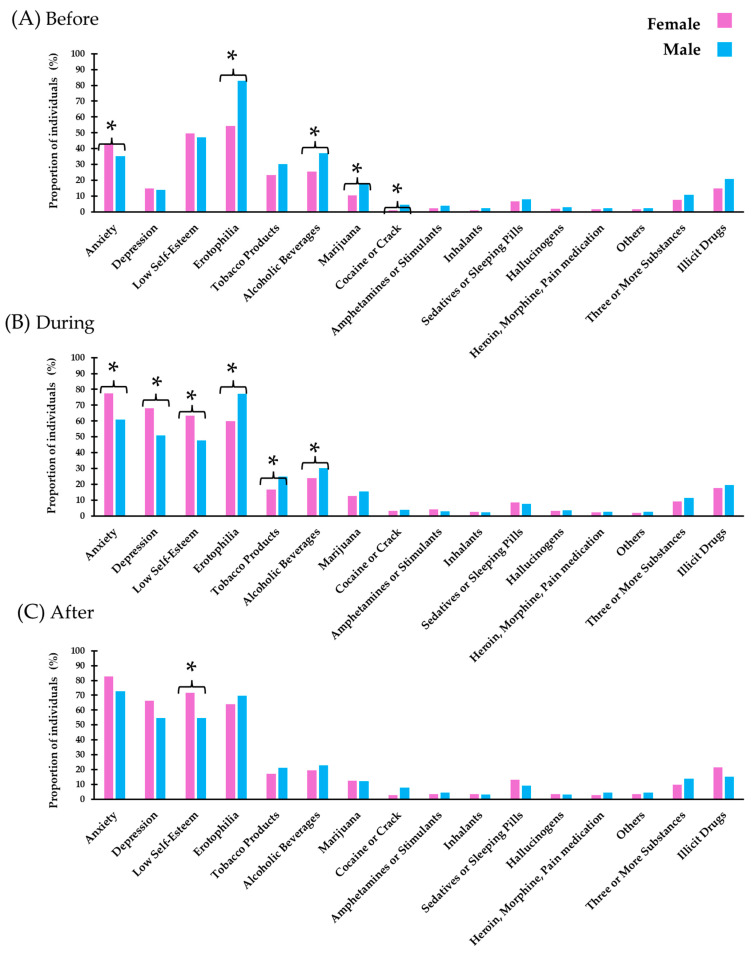
Gender Differences in Mental Health Indicators and Substance Use Among University Students Before (**A**), During (**B**), and After (**C**) the COVID-19 Pandemic. * Female vs. male students were compared using Fisher’s exact test. Anxiety and Depression were assessed according to the Hospital Anxiety and Depression Scale (HADS). The Rosenberg Self-Esteem Scale (score range: 10 to 40) was used to measure self-esteem, with scores ≤ 29 classified as low self-esteem. Erotophilia was evaluated with the Erotic Response and Orientation Scale (EROS), with scores ≥ 60 indicating erotophilia. Substance use risk levels (moderate/high) were determined using the Alcohol, Smoking, and Substance Involvement Screening Test (ASSIST) [51]. Illicit drugs include substances under international control that may or may not have legitimate medical uses but are produced, trafficked, or consumed outside legal frameworks. These include marijuana, cocaine or crack, amphetamines or stimulants, inhalants, sedatives or sleeping pills, hallucinogens, heroin, morphine, and pain medication.

While Table 5 provides detailed numerical data, the figure offers a clearer visualization of trends and differences between groups. Figure 2 allows for an easier comparison of changes in mental health and substance use across “before”, “during”, and “after” the pandemic, as well as a direct comparison between female and male students, making it simpler to observe the temporal shifts and gender disparities across these variables. Table 5 and Figure 2 present data that highlight gender differences in anxiety, depression, self-esteem, and moderate/high risk of addictions before, during, and after the pandemic. Figure 2 shows that anxiety levels were consistently higher in females than in males across all periods, with the most significant difference occurring during the pandemic (77.5% in females vs. 60.9% in males). Depression was also more prevalent in females during and after the pandemic, with 68.0% of females experiencing depression during the pandemic compared to 50.9% of males. In terms of self-esteem, females had lower self-esteem scores than males during and after the pandemic, with a notable increase in low self-esteem among females after the pandemic (71.7% in females vs. 54.5% in males). For erotophilia, males showed higher levels than females before and during the pandemic, but the difference was less pronounced after the pandemic. Regarding substance use, males had higher percentages of moderate/high risk for alcohol and tobacco use compared to females before and during the pandemic. However, the difference between genders in these behaviors became less significant after the pandemic. For example, the use of marijuana, cocaine, and other substances remained relatively stable across the periods, with males generally showing higher usage rates than females.

Table 6 presents the key factors associated with anxiety and depression among university students before and during/after the pandemic, using a multivariate logistic regression analysis. Before the pandemic, factors such as older age, anxiety, and low self-esteem were strongly linked to a higher risk of depression, while amphetamine use appeared to reduce this risk. During and after the pandemic, the relationship between anxiety, low self-esteem, and depression intensified, with sedative use emerging as a new risk factor associated with depression. On the other hand, erotophilia and car ownership acted as protective factors against depression during this period.

Regarding anxiety, being female, having depression, and low self-esteem were significant risk factors before the pandemic. After the pandemic, depression and low self-esteem remained strongly associated with anxiety, while car ownership continued to serve as a protective factor. In addition to the latter factor, Table 6 highlights that older age increased the likelihood of depression before the pandemic (AdOR 1.38, 95% CI 1.07–1.77, *p* = 0.012). Anxiety was a strong predictor of depression both before (AdOR 3.78, 95% CI 1.73–8.26, *p* = 0.001) and during/after the pandemic (AdOR 5.11, 95% CI 3.10–8.42, *p* < 0.001). Low self-esteem was consistently associated with the risk of depression before (AdOR 5.35, 95% CI 2.53–11.30, *p* < 0.001) and during/after the pandemic (AdOR 6.9, 95% CI 4.33–10.87, *p* < 0.001).

Regarding illicit substance use, Table 7 presents the results of a multivariate logistic regression analysis identifying factors associated with moderate/high risk for illicit substance use before and during/after the COVID-19 pandemic. 

Moderate/high risk for illicit substance consumption was strongly associated with tobacco use both before and during/after the pandemic (AdOR 5.67, 95% CI 2.74–11.70; and AdOR 7.65, 95% CI 4.57–12.82, respectively) (Table 7). Erotophilia was also associated with illicit substance use during both periods of this analysis. Additionally, alcohol consumption (AdOR 2.41, 95% CI 1.44–4.05) and anxiety (AdOR 1.92, 95% CI 1.07–3.46) were factors associated with illicit substance use only during/after the pandemic (Table 7).

This analysis revealed that anxiety was associated with a higher risk for illicit substance use only during/after the pandemic (AdOR 1.92, 95% CI 1.07–3.46, *p* = 0.029). Erotophilia was a significant factor in both periods, with adjusted odds ratios of 3.53 (95% CI 1.41–8.80, *p* < 0.001) before and 2.04 (95% CI 1.10–3.79, *p* = 0.023) during/after the pandemic. Tobacco products consistently showed a strong association with illicit substance use risk, with AdORs of 5.67 (95% CI 2.74–11.70, *p* < 0.001) before and 7.65 (95% CI 4.57–12.82, *p* < 0.001) during/after the pandemic. Alcoholic beverages were associated with illicit substance use only during/after the pandemic (AdOR 2.41, 95% CI 1.44–4.05, *p* = 0.001), but not before.

Table 8 provides a comprehensive overview of the proportion of students identified as being at high risk for substance use and probable dependence before, during, and after the COVID-19 pandemic. The overall prevalence of high-risk for any substance use among students was 4.9%, 5.1%, and 4.3% before, during, and after the pandemic, respectively. The data indicate a slight increase in tobacco use (from 1.3% to 1.9%) and sedatives (from 0.3% to 1.0%) post-pandemic, suggesting that these substances may have been used more as coping mechanisms for stress and anxiety. Conversely, alcohol consumption decreased significantly (from 4.3% to 1.4%) after the pandemic, likely due to a reduction in social activities that typically encourage alcohol use. Marijuana use remained stable, while there was a slight increase in cocaine and other substance consumption after the pandemic, potentially reflecting reduced access during lockdowns and a subsequent rebound.

## 4. Discussion

Depression and anxiety are critical mental health concerns, particularly among young people. The results of this study reveal a significant impact of the COVID-19 pandemic on mental health and substance use patterns among university students. The observed increases in anxiety and depression levels during and after the pandemic underscore the pandemic’s profound effects on student well-being. Although contracting the illness, being hospitalized due to COVID-19, or experiencing the loss of a family are commonly cited causes for depression and anxiety, this factor was analyzed in this study and found to be non-significant. This suggests that these psychological disorders may be influenced more by factors beyond the direct impact of the disease or risk of infection, such as the social environment and prolonged isolation. These findings highlight the vulnerability of young adults to contextual changes and external stressors, such as a global pandemic [16]. 

In the context of mental health during the COVID-19 pandemic, multiple studies have addressed the effects of the pandemic on anxiety and substance use. For example, a study by Xiong et al. (2020) [59] found that the prevalence of anxiety and substance use increased significantly during the pandemic, with a particularly high impact on young people and those with a history of mental health problems [59,60]. Similarly, Czeisler et al. (2020) reported an increase in drug use and anxiety symptoms among U.S. adults during the pandemic [61]. Our study aligns with these findings, showing that the pandemic had a significant impact on anxiety levels and substance use in our study population. However, unlike some studies that focused on specific populations or developed countries [62,63], our research provides valuable data on a diverse population, including varied demographic variables and socioeconomic contexts across three periods of COVID transition. 

The most notable result is that the high levels of depression and anxiety did not return after the pandemic to pre-pandemic levels and remained high. Before the pandemic, the proportion of students with anxiety was 40.0%, which increased to 71.7% during the pandemic and to 79.6% after the pandemic. Similarly, depression increased from 14.4% to 61.9% during the pandemic, remaining at 62.6% after the pandemic. These increases could be associated with such factors as social isolation, economic uncertainty, and concerns about personal and loved ones’ health [16,18]. While multilevel regression analysis could provide a deeper understanding of variations in mental health outcomes due to contextual factors and individual characteristics, we focused our analysis on the relationship between substance use and its effects on students across the pandemic periods. Future studies may benefit from incorporating multilevel approaches to capture the influence of additional variables more comprehensively. Self-esteem was also affected, with a significant decrease during and after the pandemic compared to the pre-pandemic period. Furthermore, delving deeper into the impact of pandemic-specific restrictions is crucial for understanding their influence on students’ emotional well-being. Assessing how factors such as lockdowns, social distancing, and limitations on social interactions affected mental health—beyond the change in educational format—could provide valuable insights for designing effective future interventions.

### 4.1. Gender Differences in Anxiety and Depression Among University Students

It is important to highlight that gender differences indicated that females were more likely to experience anxiety and low self-esteem both during and after the pandemic, suggesting greater vulnerability in this group. Several factors may contribute to this heightened vulnerability among young females. At this age, university students often face intense academic pressures, social expectations, and a significant transition to adulthood [64,65]. The COVID-19 pandemic introduced additional stressors, including disruptions to their education, social isolation, and uncertainty about the future. Young females may also encounter societal pressures regarding appearance, relationships, and academic performance, which can exacerbate feelings of anxiety and inadequacy [66,67,68]. Furthermore, the emotional burden of supporting friends and family during challenging times may lead to increased stress, highlighting the need for targeted mental health resources [67]. Conversely, males showed a higher proportion of erotophilia before and during the pandemic. This may reflect cultural norms that encourage males to openly express sexuality while discouraging emotional vulnerability. Such norms can also influence substance use behaviors, with males potentially using alcohol as a means of asserting social status or coping with stress [69,70]. However, our study extends this by providing post-pandemic data, which is often missing in other studies. The trends observed in our research suggest a complex interplay of gender, societal expectations, and the evolving landscape of substance use among young university students during and after the pandemic, warranting further exploration in future studies.

### 4.2. Substance Use Among University Students

The analysis of substance use revealed notable patterns: while alcohol and tobacco use decreased during and after the pandemic, there was a rise in the use of sedatives or sleeping pills. Specifically, the proportion of students at moderate/high risk for alcohol addiction decreased from 29.9% before the pandemic to 26.2% during and 20.2% after the pandemic. Similarly, the risk of tobacco addiction fell from 26.0% before the pandemic to 19.6% during and 18.2% after the pandemic. In contrast, the use of sedatives or sleeping pills increased from 7.1% before the pandemic to 11.7% after the pandemic. These findings align with the existing literature that shows a reduction in tobacco and alcohol use during the pandemic [71,72]. However, our study extends this by providing post-pandemic data, which is often missing in other studies. This allows for a more comprehensive understanding of substance use trends as they evolve beyond the pandemic period. 

Furthermore, future studies should delve deeper into alcohol consumption by considering not only the frequency of use but also the type of alcohol and the quantity consumed. This nuanced understanding is vital in exploring the self-medication practices among university students. Despite these considerations, our findings indicate that alcohol continues to be used as a means of escape, highlighting the need for targeted interventions and support systems for students struggling with mental health issues. Additionally, future research should consider investigating the use of electronic cigarettes containing various substances, as these could potentially trigger other health conditions [73]. This exploration is particularly important given the rise in popularity of e-cigarettes among young adults, which may contribute to different patterns of substance use and associated health risks [15].

### 4.3. Socioeconomic Factors and the Role of Car Ownership Against Anxiety and Depression 

In line with the points mentioned above, the relationship between socioeconomic status and access to healthcare is a crucial factor in studying young people’s mental health. As noted, while all students in Mexico have access to free public healthcare, higher socioeconomic status often enables additional access to mental health services that are less readily available in the public system [45]. However, this study did not analyze healthcare service usage or out-of-pocket health expenses, which future research should consider.

Our findings show that most participants came from middle socioeconomic backgrounds (51.8%), with 36.2% from higher-income groups and 12% from vulnerable or low-income levels [27]. Our data are consistent with national statistics in Mexico, where the middle class comprises approximately 42% of the population, and those living in poverty account for about 22%. A deeper exploration of socioeconomic factors may reveal complexities that were beyond this study’s scope. Socioeconomic status likely influences both healthcare access and access to substances, licit or illicit. 

Key socioeconomic factors—such as educational access, financial resources, and income—could either elevate or mitigate mental health risks. Although these factors were not included in this study, future research should explore them further, as they could act as triggers in various life situations. Additionally, examining whether students have responsibilities such as being parents, primary earners, or employment status, and even how they manage academic schedules or vacations, may shed light on how access to resources and stress levels impact mental health in young adults. Taking into account substance use, students from higher socioeconomic levels may have easier access to prescription medications, including those used for anxiety or depression, and a higher likelihood of purchasing these substances without prescription [74]. Conversely, those from lower socioeconomic levels might encounter greater barriers in accessing medical treatment, potentially leading to the use of non-prescribed or illegal substances as coping mechanisms [75]. This divergence could contribute to differing mental health outcomes, which future research should explore more thoroughly. 

One of the variables of particular interest is that the results suggest that car ownership, potentially facilitating greater mobility during the pandemic, acted as a protective factor (Table 6) against depression. Previous studies have demonstrated that car ownership increased self-esteem among university students [45,76]. This study aided with reduced depression risk, complementing findings that link automobile ownership to enhanced sexual self-esteem and overall well-being. As observed, the lower proportion of students may be influenced by the possession of assets or services, such as car ownership, which likely impacts mental health. The results obtained are in line with previous studies that suggest that automobile ownership among university students may mitigate feelings of isolation, promote self-esteem, and enhance sexual self-esteem—variables previously associated with lower levels of depression [77]. The psychological benefits of car ownership, such as increased self-efficacy and empowerment, are also important contributors to mental well-being. These results highlight the need for further research into the interaction of socioeconomic factors, including car ownership, with other markers like income level and access to resources to better understand their influence on mental health. 

### 4.4. Addressing Mental Health Challenges: Strategies for Support and Prevention

This study’s findings underscore the need for targeted interventions to address the mental health of university students, especially in times of crisis [78,79]. Strengthening psychological support services and promoting healthy coping strategies are essential to mitigating the negative effects of anxiety and depression [80]. Additionally, the decrease in alcohol and tobacco use might indicate a shift in coping mechanisms, suggesting the need to monitor and manage the use of sedatives and other potentially problematic medications. Universities and public policies should focus on creating supportive environments and reducing the stigma associated with seeking help for mental health and substance use issues. Implementing educational and early intervention programs can be crucial in improving the overall well-being of university students. Given that these data reflect behaviors in young adults, it is crucial that educational and preventive programs address these substance use patterns [81,82]. Educational programs should focus on preventing tobacco, alcohol, and drug use, highlighting the risks associated with prolonged use and its impact on mental and physical health. The pandemic has exacerbated anxiety and stress in young adults, which can lead to substance use as a coping mechanism. It is vital to include stress management strategies and psychological support in educational programs.

Providing access to resources for the treatment and prevention of substance use, including helplines, counseling, and rehabilitation programs, can be crucial in reducing long-term substance use. Detecting and addressing substance use at early stages is fundamental. Educational programs should include early detection components and referral to specialized services. Recognizing that the pandemic has changed substance use patterns, programs should adapt to address these new trends and provide adequate support in the post-pandemic context. Additionally, considering new strategies to evaluate teaching methods and proposing new approaches to include in current educational models could help create more resilient students.

### 4.5. Addressing Mental Health Challenges: Impact in Mexico

The COVID-19 pandemic also had a significant impact on the school context in Mexico [63,83]. The suspension of in-person classes and the transition to online education presented numerous challenges for both students and teachers [83]. Many students faced difficulties accessing technological resources and connectivity, exacerbating pre-existing educational inequalities [63]. Moreover, the lack of social interaction and changes in school routines contributed are related to an increase in anxiety and stress among students.

Teachers, on the other hand, had to quickly adapt to new teaching platforms and pedagogical methods, which created an additional workload and stress [84]. Emotional and psychological support for both students and teachers became crucial in addressing the challenges of education during the pandemic [85]. In this context, it is essential to develop strategies and educational policies that not only mitigate the pandemic’s impact but also strengthen the resilience and well-being of the school community in Mexico.

In previous studies focusing on health students in Mexico, levels of depression, anxiety, and stress have been determined to be 27%, 56%, and 5%, respectively [62,86]. These findings highlight the significant mental health challenges faced by students pursuing health-related fields. Given the unique stressors associated with health professions [87], it would be valuable to evaluate these parameters in future research among students from different academic disciplines. Such an approach may help identify whether certain fields of study are associated with higher levels of mental health issues and guide the implementation of tailored interventions to support students in those specific areas.

In comparing our findings with those from neighboring countries and nations of similar income levels, we observe both agreements and disagreements regarding the prevalence of anxiety and substance use among university students. For instance, studies from Latin American countries have reported similar trends of increased anxiety, violence against females, and altered substance use patterns during the COVID-19 pandemic, highlighting a regional challenge that calls for targeted interventions [88,89]. However, discrepancies arise when examining data from high-income countries, where the patterns of substance use have often been reported as more stable, potentially reflecting differences in social support systems and healthcare access [90,91]. These insights underscore the need for culturally sensitive approaches tailored to the unique challenges faced by students in different socioeconomic contexts. Clinically, the implications of these findings suggest that mental health services and substance use interventions must be adapted to address the specific needs of university students in diverse settings [92,93,94]. Future research should focus on longitudinal studies that track mental health and substance use trends post-pandemic, comparing these patterns across various demographic and economic contexts to better understand the long-term impacts and inform effective interventions.

### 4.6. Limitations of This Study

One of the limitations of this study is that it was conducted within a traditional school system, leaving out other forms of education, such as semi-scholastic systems, which represent a considerable percentage of university programs in Mexico [95]. These systems could involve additional variables, such as tuition payment or the diverse teaching methods implemented before, during, and after the pandemic [85,96]. In addition to this limitation of this study is the lack of differentiation between the justified and excessive use of prescribed and non-prescribed medications, particularly in relation to the use of sedatives and their association with anxiety and depression. While we classified substances as non-prescribed for the purpose of this research, we recognize the importance of distinguishing the type of medication used. 

Additionally, a limitation of this study is the use of a non-probability sampling design, which may limit the generalizability of the findings to the broader population of university students. Since participants were selected based on availability, there is a risk of selection bias. This limitation could affect the representativeness of the sample, potentially reducing the applicability of the results to different contexts or populations. 

Finally, another limitation of this study is the specific academic context in which it was conducted and the data collection methods employed (in-person vs. online). These factors may have influenced participants’ responses, potentially impacting the validity of the findings. For example, collecting data online could have affected participants’ comfort and openness, possibly leading to variations in their reported experiences [97]. Nevertheless, given the circumstances of the pandemic, online data collection was an effective approach to ensuring participant safety while gathering necessary information. It is also worth noting that while the instruments used in this study do not precisely reflect the participants’ current psychological state, they provide valuable insights. These findings could inform future measures and support further data collection through different instruments or methods, ideally with the involvement of a healthcare professional.

### 4.7. Perspectives and Strengths of This Study and Possible Strategies for Anxiety and Depression 

One factor worth exploring in future studies is whether the consumption of certain plant species legally distributed with effects on the central nervous system (such as teas, infusions, preparations, or tonics) increased during this period. Additionally, the use of cannabis extracts for treating symptoms of anxiety and depression has been reported, although it was not considered in this research. While cannabis is often deemed non-toxic, there have been reports of patients experiencing diaphoresis due to abuse during the pandemic period, which could be linked to these psychological conditions [98]. Notably, during the COVID-19 pandemic, the use of herbs for symptoms associated with anxiety and depression increased among the Mexican population [99]. Future research should include an analysis of alternative therapies that may have been employed, such as Ayahuasca (*Banisteriopsis caapi*) [100], Peyote (*Lophophora williamsii*) [101], hallucinogenic mushrooms (e.g., *Psilocybe* spp.) [102], and other traditional herbal medicines. These substances, often used either as self-medication or suggested by practitioners of alternative therapies, have been reported with growing frequency in Mexico [103]. 

Future research could build on these findings by exploring the effects of different educational modes, the role of social support, and the long-term consequences of the pandemic on mental health and substance use. Additionally, considering new strategies for assessing teaching methods and making proposals to include in current educational models could generate trends toward creating more resilient students. Considering these challenges, we recommend that Mexican universities establish comprehensive mental health programs that provide accessible resources and support for both students and faculty. This may include on-campus counseling services, mental health workshops, and training for teachers to recognize and address mental health issues among students.

This study presents several strengths that contribute to its value in understanding the impact of the COVID-19 pandemic on university students’ mental health and substance use patterns. One notable strength is the comprehensive analysis of changes in anxiety, depression, and substance use before, during, and after the pandemic, providing a detailed picture of the pandemic’s effects over time. The inclusion of a wide range of substances and the use of multivariate logistic regression to identify associated factors offers a nuanced understanding of the relationship between mental health issues and substance use. Additionally, this study highlights significant findings related to the increase in tobacco and sedative use and the decrease in alcohol consumption, suggesting potential coping mechanisms and changes in behavior. This research also considers demographic factors and provides insights into gender differences in mental health and substance use, enhancing the relevance of the findings. Moreover, this study’s focus on a diverse student population in Mexico adds valuable context to the global discussion on pandemic-related health impacts. Future research could build on these findings by exploring the effects of different educational modes, the role of social support, and the long-term consequences of the pandemic on mental health and substance use. 

One strategy for the educational programs might be implementing peer support systems that might foster a sense of community and belonging, which is critical for student well-being. Furthermore, universities should develop substance use prevention programs that specifically address the use of drugs and alcohol, providing education on the risks associated with substance use and promoting healthier coping strategies. These programs can be complemented by support services for students struggling with substance use issues, ensuring a holistic approach to students’ health and wellness.

It is important to emphasize that there are many more factors to explore in this context. Additional studies are necessary to raise awareness about the complexities of mental health outcomes, as we are inherently social beings. While the effects of restrictive measures may have had some positive impacts, prolonged exposure to these factors has led to significant mental health challenges. Furthermore, future research could investigate trends among younger individuals who may demonstrate lower resilience, higher levels of stress and anxiety, and a significant generational shift that will require us to adopt new strategies for intervention in all aspects of society. 

## 5. Conclusions

Considering the results, it is evident that the COVID-19 pandemic has left a significant impact on the mental health and substance use patterns of university students. The persistent increases in anxiety and depression levels, which have not returned to pre-pandemic baselines, highlight the vulnerability of this population to contextual changes and external stressors. The observed reduction in alcohol and tobacco use, coupled with an increase in sedative or sleep aid usage, suggests a shift in coping mechanisms, potentially as a response to prolonged stress and anxiety.

These findings, along with observed gender differences, underscore the importance of targeted, evidence-based interventions. Universities and policymakers should focus efforts on creating supportive environments that prioritize mental health. Recommendations include developing adaptive mental health policies that recognize the specific needs of students, implementing counseling services, and establishing accessible, stigma-free mental health resources on campus. Additionally, promoting healthy coping strategies and prevention programs that address changes in substance use and mental health trends post-pandemic is crucial. Proactive measures could involve workshops, peer support programs, and educational campaigns designed to help students build resilience, manage stress effectively, and make informed choices about substance use.

## Figures and Tables

**Table 1:** Main characteristics of emerging adults are university students.

Characteristic	All (*n* = 2167)
Gender	
Female	65.1%
Male	34.9%
Age (years)	19.7 ± 1.4
BMI	24.1 ± 6.6
University level (semester)	3.7 ± 2.3
Mean grade (0–10)	8.7 ± 0.8
Has begun sexual activity	60.7%
FSI age (years)	17.2 ± 1.8
Number of sexual partners	2.9 ± 3.3
Has child/children	1.7%
Have a car	18.5%
Socioeconomic level	
High	36.2%
Middle level	51.8%
Vulnerable middle-class/poor	12.0%

BMI: Body Mass Index; FSI: Age at first sexual intercourse (vaginal/anal); Socioeconomic level: According to the 2022 guidelines of the Mexican Association of Market Intelligence and Opinion Agencies (AMAI), households are classified into three groups: A/B (high); C (middle level); and D/E (vulnerable middle-class/poor).

**Table 2:** Comparison of Anxiety, Depression, Self-Esteem (Rosenberg), and Erotic Response and Sexual Orientation Scale Scores (EROS) in Emerging Adult University Students by Pandemic Period.

	Before	During	After	Inter-Groups	Before vs. During	During vs. After	Before vs. After
Anxiety	6.9 (6.7–7.1)	10.2 (9.9–10.4)	10.8 (10.3–11.4)	<0.001	<0.001	0.043	<0.001
Depression	3.5 (3.4–3.7)	7.9 (7.7–8.2)	7.5 (7.0–8.0)	<0.001	<0.001	0.273	<0.001
Rosenberg scale	29.9 (29.6–30.2)	28.2 (27.8–28.5)	27.7 (27.9–28.3)	<0.001	<0.001	0.627	<0.001
EROS	67.6 (65.4–69.7)	68.3 (66.6–69.9)	67.3 (64.5–70.0)	0.760	0.999	0.999	0.999

Values are presented as mean and 95% CI. Anxiety and Depression, according to Anxiety and Depression Scale (HADS). Clinical cut-off points are ≥8 for anxiety and ≥7 for depression. The Rosenberg Self-Esteem Scale scores range from 10 to 40, with low self-esteem classified as a score of 29 or lower. EROS: Erotic Response and Sexual Orientation Scale, with a score of 60 or higher indicating erotophilia.

**Table 3:** Proportion of Young Adults with Anxiety, Depression, Low Self-Esteem, Erotophilia, and Risk for Addictions Before, During, and After the Pandemic.

	Pandemic Period			*p* *			
	Before *n* = 1179	During *n* = 779	After *n* = 209	Inter-Groups	Before vs. During	During vs. After	Before vs. After
	100.0%	100.0%	100.0%				
Anxiety (HADS)	40.0%	71.7%	79.6%	<0.001	<0.001	0.020	<0.001
Depression (HADS)	14.4%	61.9%	62.6%	<0.001	<0.001	0.940	<0.001
Low Self-Esteem	48.5%	57.9%	66.5%	<0.001	<0.001	0.030	<0.001
Erotophilia	65.5%	65.9%	65.9%	0.993	0.477	0.533	0.503
Moderate/high risk by							
Tobacco products	26.00%	19.60%	18.20%	0.014	0.005	0.697	0.032
Alcoholic beverages	29.9%	26.2%	20.2%	0.024	0.154	0.043	0.009
Marijuana	13.40%	13.50%	12.20%	0.876	0.513	0.651	0.713
Cocaine or Crack	2.40%	3.40%	4.20%	0.434	0.394	0.538	0.228
Amphetamines **	2.90%	3.70%	3.80%	0.731	0.517	0.542	0.635
Inhalants	1.60%	2.50%	3.30%	0.325	0.314	0.484	0.156
Pills ***	7.1%	8.1%	11.7%	0.140	0.581	0.104	0.061
Hallucinogens	2.2%	3.3%	3.3%	0.530	0.379	0.595	0.438
Heroin ****	1.8%	2.3%	3.3%	0.498	0.682	0.456	0.265
Others	1.8%	2.2%	3.7%	0.319	0.834	0.214	0.174
Illicit Drugs	17.2%	18.2%	19.2%	0.802	0.698	0.765	0.516
3 > Substances	8.8%	9.7%	10.8%	0.714	0.613	0.699	0.476

* Fisher exact test. HADS: Hospital Anxiety and Depression Scale, with scores ranging from 0 to 21 for depressive symptoms and 0 to 21 for anxiety. Clinical cut-offs were set ≥8 for the presence of anxiety and ≥7 for depression. The Rosenberg Self-Esteem Scale has scores ranging from 10 to 40, with low self-esteem classified as a score of 29 or lower. A moderate/high risk of different addictions was determined by the Alcohol, Smoking, and Substance Involvement Screening Test (ASSIST) [51]. Illicit drugs are those under international control, which may or may not have legitimate medical use but are produced, trafficked, and/or consumed outside the legal framework (Marijuana, Cocaine or Crack, Amphetamines or Stimulants, Inhalants, Sedatives or Sleeping Pills, Hallucinogens, Heroin, Morphine, Pain Medication). ** Amphetamines or Stimulants. *** Sedatives or Sleeping Pills. **** Heroin, Morphine, or Pain medication.

**Table 4:** Impact of Socioeconomic Level and Car Ownership on Anxiety and Depression During the COVID-19 Pandemic.

	Pandemic Period				Pandemic Period			
	Before		During/After		Before		During/After	
	Anxiety		Anxiety		Depression		Depression	
	Yes	No	Yes	No	Yes	No	Yes	No
Socioeconomic level								
High	32.3%	67.7%	71.1%	28.9%	12.90%	87.10%	53.40%	46.60%
Middle	41.6%	58.4%	75.7%	24.3%	13.30%	86.70%	67.60%	32.40%
Vulnerable middle-class/poor	38.9%	61.1%	70.0%	30.0%	14.50%	85.50%	64.20%	35.80%
*p*-value *	0.444		0.208		0.863		0.001	
Automobile								
Yes	36.00%	64.00%	61.9%	38.1%	9.0%	91.0%	45.80%	54.20%
No	41.3%	58.7%	75.4%	24.6%	16.0%	84.0%	65.10%	34.90%
*p*-value **	0.058		<0.001		<0.001		<0.001	

* Fisher’s exact test comparing the proportions of anxiety or depression among the three socioeconomic levels during each pandemic time, using 2 × 3 contingency tables. ** Fisher’s exact test comparing the proportions of anxiety or depression between those who own a car versus those who do not within the same pandemic time, using 2 × 2 contingency tables.

**Table 5:** Difference between genders in anxiety, depression, self-esteem, and moderate/high risk for addictions before, during, and after the pandemic.

	Female	Male	*p* *		Female	Male	*p* *
Anxiety				Depression			
Before	42.80%	35.10%	0.007	Before	14.70%	13.90%	0.683
During	77.50%	60.90%	<0.001	During	68.00%	50.90%	<0.001
After	82.80%	72.70%	0.100	After	66.20%	54.50%	0.125
Low Self-Esteem				Erotophilia			
Before	49.50%	47.10%	0.420	Before	54.20%	82.70%	<0.001
During	63.50%	47.70%	<0.001	During	59.80%	77.10%	<0.001
After	71.70%	54.50%	0.018	After	64.10%	69.70%	0.531
Tobacco Products				Alcoholic Beverages			
Before	23.20%	30.20%	0.130	Before	25.40%	37.20%	0.008
During	16.80%	24.70%	0.009	During	24.00%	30.10%	0.039
After	17.20%	21.20%	0.567	After	19.30%	22.70%	0.584
Marijuana				Cocaine or Crack			
Before	10.30%	18.20%	0.017	Before	1.10%	4.40%	0.032
During	12.50%	15.40%	0.277	During	3.10%	3.90%	0.544
After	12.40%	12.10%	1.000	After	2.80%	7.60%	0.142
Amphetamines or Stimulants				Inhalants			
Before	2.20%	3.90%	0.391	Before	1.10%	2.20%	0.446
During	4.10%	2.90%	0.434	During	2.70%	2.20%	0.813
After	3.40%	4.50%	0.707	After	3.40%	3.00%	0.999
Sedatives or Sleeping Pills				Hallucinogens			
Before	6.70%	7.80%	0.710	Before	1.90%	2.80%	0.534
During	8.40%	7.50%	0.785	During	3.10%	3.60%	0.835
After	13.10%	9.10%	0.495	After	3.40%	3.00%	1.000
Heroin, Morphine, Pain medication				Others			
Before	1.50%	2.20%	0.719	Before	1.50%	2.20%	0.719
During	2.10%	2.50%	0.805	During	2.00%	2.50%	0.614
After	2.80%	4.50%	0.680	After	3.40%	4.50%	0.707
Three or More Substances				Illicit Drugs			
Before	7.70%	10.70%	0.307	Before	14.90%	20.80%	0.124
During	9.00%	11.20%	0.379	During	17.60%	19.40%	0.563
After	9.70%	13.60%	0.475	After	21.40%	15.20%	0.350

* Female vs. male, Fisher exact test. Anxiety and Depression, according to Anxiety and Depression Scale (HADS). The Rosenberg Self-Esteem Scale has a score from 10 to 40, and low self-esteem was classified as a score equal to or lower than 29. Erotophilia was considered if a score of 60 or higher was obtained on the EROS scale (Erotic Response and Sexual Orientation Scale). A moderate/high risk of different addictions was determined by the Alcohol, Smoking, and Substance Involvement Screening Test (ASSIST) [51]. Illicit drugs were defined as those under international control that may or may not have legitimate medical use but are produced, trafficked, and/or consumed outside the legal framework (Marijuana, Cocaine or Crack, Amphetamines or Stimulants, Inhalants, Sedatives or Sleeping Pills, Hallucinogens, Heroin, Morphine, Pain Medication).

**Table 6:** Multivariate Logistic Regression Analysis for Detecting Factors Associated with Depression or Anxiety Before and During/After the COVID-19 Pandemic.

Risk Factors Associated with Depression	Before				During/After			
	AdOR	95% CI		*p*	AdOR	95% CI		*p*
Age	1.38	1.07	1.77	0.012				
Anxiety	3.78	1.73	8.26	0.001	5.11	3.10	8.42	0.000
Low Self-Esteem	5.35	2.53	11.30	0.000	6.9	4.33	10.87	0.000
Sedatives or Sleeping Pills Use					2.15	1.12	4.14	0.021
Amphetamines	0.37	0.14	0.95	0.040	0.44	0.17	1.15	0.093
Erotophilia					0.48	0.28	0.83	0.008
Car Ownership					0.51	0.29	0.91	0.023
**Risk Factors Associated with Anxiety**	**Before**				**During/After**			
	**AdOR**	**95% CI**		***p***	**AdOR**	**95% CI**		***p***
Female	1.64	1.01	2.67	0.047				
Depression	4.04	1.84	8.83	0.000	4.87	2.98	7.95	0.000
Low Self-Esteem	3.68	2.05	6.61	0.000	3.29	2.01	5.38	0.000
Car Ownership					0.56	0.33	0.97	0.040

Multivariate binary logistic regression analysis was performed to obtain adjusted odds ratios (AdOR) with their 95% confidence intervals (CI) and *p*-values. The most parsimonious multivariate model was selected using the backward stepwise selection method, with a probability threshold of 0.15 for variable entry and 0.25 for variable elimination. The variables included were age, gender (female), initiation of sexual activity, initiation of sexual activity at age 17 or younger, car ownership, low self-esteem, depression, anxiety, erotophilia, and moderate/high risk for addictions to tobacco products, alcoholic beverages, marijuana, cocaine or crack, amphetamines or stimulants, inhalants, sedatives or sleeping pills, hallucinogens, heroin, morphine, or pain medications. During/after the pandemic period, additional variables included contracting COVID-19, hospitalization or death of a household member, and COVID-19-related worry (rated 9/10 on a 1–10 scale).

**Table 7:** Multivariate Logistic Regression for Detecting Factors Associated with Moderate/High Risk for Illicit Substance Addiction Before and During/After the COVID-19 Pandemic.

	Before				During/After			
	AdOR	95% CI		*p*	AdOR	95% CI		*p*
Anxiety					1.92	1.07	3.46	0.029
Erotophilia	3.53	1.41	8.80	<0.001	2.04	1.10	3.79	0.023
Tobacco products	5.67	2.74	11.70	<0.001	7.65	4.57	12.82	<0.001
Alcoholic beverages	1.74	0.84	3.59	0.133	2.41	1.44	4.05	0.001

Multivariate binary logistic regression analysis was conducted to obtain adjusted odds ratios (AdOR) with their 95% confidence intervals (CI) and *p*-values. The variables for the most parsimonious multivariate model were selected using the backward stepwise selection method, with a probability threshold of 0.15 for variable entry and 0.25 for elimination. The included variables were age, gender (female), initiation of sexual activity, initiation of sexual activity at age 17 or younger, car ownership, low self-esteem, depression, anxiety, erotophilia, and moderate/high risk for addictions to tobacco products, alcoholic beverages, marijuana, cocaine or crack, amphetamines or stimulants, inhalants, sedatives or sleeping pills, hallucinogens, heroin, morphine, or pain medications. During/after the pandemic period, additional variables included contracting COVID-19, hospitalization or death of a household member, and COVID-19-related worry (rated 9/10 on a 1–10 scale).

**Table 8:** Proportion of Students at High Risk and Probable Dependence on Substances Before, During, and After the Pandemic.

	Pandemic Period		
	Before *n* = 1179	During *n* = 779	After *n* = 209
Tobacco products	1.30%	1.70%	1.90%
Alcoholic beverages	4.30%	4.10%	1.40%
Marijuana	0.50%	0.40%	0.50%
Cocaine or Crack	0.30%	0.00%	0.50%
Amphetamines or Stimulants	0.00%	0.00%	0.50%
Inhalants	0.00%	0.00%	0.50%
Sedatives or Sleeping Pills	0.30%	0.60%	1.00%
Hallucinogens	0.30%	0.00%	0.50%
Heroin, Morphine, Pain medication	0.30%	0.00%	0.50%
Others	0.30%	0.10%	0.50%
Any Substance	4.90%	5.10%	4.30%

Substances included are tobacco products, alcoholic beverages, marijuana, cocaine or crack, amphetamines or stimulants, inhalants, sedatives or sleeping pills, hallucinogens, heroin, morphine, and pain medications. Percentages for “Any Substance” represent the proportion of students at high risk for one or more substances. Data sources: self-reported surveys conducted at three time points: before; during; and after the pandemic.

## Data Availability

The datasets used and/or analyzed during the current study are available from the corresponding author upon reasonable request.
